# Publisher Correction: Agonist-antagonist muscle strain in the residual limb preserves motor control and perception after amputation

**DOI:** 10.1038/s43856-022-00188-3

**Published:** 2022-09-26

**Authors:** Hyungeun Song, Erica A. Israel, Samantha Gutierrez-Arango, Ashley C. Teng, Shriya S. Srinivasan, Lisa E. Freed, Hugh M. Herr

**Affiliations:** 1 K. Lisa Yang Center for Bionics, Massachusetts Institute of Technology, Cambridge, MA USA; 2 Harvard-MIT Division of Health Sciences and Technology, Massachusetts Institute of Technology, Cambridge, MA USA; 3 Mechanical Engineering Department, Massachusetts Institute of Technology, Cambridge, MA USA; 4 Harvard Medical School, Cambridge, MA USA

**Keywords:** Motor control, Peripheral nervous system

Correction to: *Communications Medicine* 10.1038/s43856-022-00162-z, published online 05 August 2022.

The original PDF version of this article contained errors in equations 3, 4 and 5. In equation 3, the second term was written as ‘${{{\rm{acos}}}}({{{{\boldsymbol{u}}}}}_{i}^{T}\cdot \frac{{{{{\boldsymbol{c}}}}}_{i}(t)}{{{{{\boldsymbol{c}}}}}_{i}(t)\parallel })$’ but should have been written as ‘${{{\rm{acos}}}}({{{{\boldsymbol{u}}}}}_{i}^{T}\cdot \frac{{{{{\boldsymbol{c}}}}}_{i}(t)}{\Vert {{{{\boldsymbol{c}}}}}_{i}(t)\Vert })$’. In equation 4, the second and third terms were written as ‘$({{{\rm{acos}}}}({{{{\boldsymbol{u}}}}}_{{{{\rm{PF}}}}}^{T}\cdot \frac{{{{{\boldsymbol{U}}}}}_{{{{\rm{S}}}}}(t)}{{{{{\boldsymbol{U}}}}}_{{{{\rm{S}}}}}(t)\parallel })-{{{\rm{acos}}}}({{{{\boldsymbol{u}}}}}_{{{{\rm{DF}}}}}^{T}\cdot \frac{{{{{\boldsymbol{U}}}}}_{{{{\rm{S}}}}}(t)}{{{{{\boldsymbol{U}}}}}_{{{{\rm{S}}}}}(t)\parallel }))$’ but should have been written as ‘$({{{\rm{acos}}}}({{{{\boldsymbol{u}}}}}_{{{{\rm{PF}}}}}^{T}\cdot \frac{{{{{\boldsymbol{U}}}}}_{{{{\rm{S}}}}}(t)}{\Vert {{{{\boldsymbol{U}}}}}_{{{{\rm{S}}}}}(t)\Vert })-{{{\rm{acos}}}}({{{{\boldsymbol{u}}}}}_{{{{\rm{DF}}}}}^{T}\cdot \frac{{{{{\boldsymbol{U}}}}}_{{{{\rm{S}}}}}(t)}{\Vert {{{{\boldsymbol{U}}}}}_{{{{\rm{S}}}}}(t)\Vert }))$’. In equation 5, the second and third terms were written as ‘$({{{\rm{acos}}}}({{{{\boldsymbol{u}}}}}_{{{{\rm{IN}}}}}^{T}\cdot \frac{{{{{\boldsymbol{U}}}}}_{{{{\rm{S}}}}}(t)}{{{{{\boldsymbol{U}}}}}_{{{{\rm{S}}}}}(t)\parallel })-{{{\rm{acos}}}}({{{{\boldsymbol{u}}}}}_{{{{\rm{EV}}}}}^{T}\cdot \frac{{{{{\boldsymbol{U}}}}}_{{{{\rm{S}}}}}(t)}{{{{{\boldsymbol{U}}}}}_{{{{\rm{S}}}}}(t)\parallel }))$’ but should have been written as ‘$({{{\rm{acos}}}}({{{{\boldsymbol{u}}}}}_{{{{\rm{IN}}}}}^{T}\cdot \frac{{{{{\boldsymbol{U}}}}}_{{{{\rm{S}}}}}(t)}{\Vert {{{{\boldsymbol{U}}}}}_{{{{\rm{S}}}}}(t)\Vert })-{{{\rm{acos}}}}({{{{\boldsymbol{u}}}}}_{{{{\rm{EV}}}}}^{T}\cdot \frac{{{{{\boldsymbol{U}}}}}_{{{{\rm{S}}}}}(t)}{\Vert {{{{\boldsymbol{U}}}}}_{{{{\rm{S}}}}}(t)\Vert }))$’. These equations have now been corrected in the PDF. The equations in the HTML version were published correctly and remain unchanged.

