# Supplementary material for: Agonist-antagonist muscle strain in the residual limb preserves motor control and perception after amputation
Source: Commun Med (Lond). 2022 Aug 5;2:97. doi: 10.1038/s43856-022-00162-z (PMC9356003; doi:10.1038/s43856-022-00162-z)
Supplement: Supplementary file 1 — Description of Additional Supplementary Files [file 43856_2022_162_MOESM1_ESM.pdf]

Description of Additional Supplementary Files

File Name: Supplementary Data 1

Description: Muscle synergy and synergy activation profiles

File Name: Supplementary Data 2

Description: Multi-DoF motor controllability

File Name: Supplementary Data 3

Description: Spatiotemporal motor controllability
