# Supplementary material for: Agonist-antagonist muscle strain in the residual limb preserves motor control and perception after amputation
Source: Commun Med (Lond). 2022 Aug 5;2:97. doi: 10.1038/s43856-022-00162-z (PMC9356003; doi:10.1038/s43856-022-00162-z)
Supplement: Supplementary file 2 — Supplementary Information [file 43856_2022_162_MOESM2_ESM.pdf]

Agonist-antagonist muscle strain in the residual limb preserves motor control and perception after amputation

Authors: Hyungeun Song[1,2*], Erica A. Israel[1], Samantha Gutierrez-Arango[1], Ashley C. Teng[1,3], Shriya S. Srinivasan[1,2], Lisa E. Freed[1], & Hugh M. Herr[1,4*]

[1] K. Lisa Yang Center for Bionics, Massachusetts Institute of Technology, Cambridge, Massachusetts, USA

[2] Harvard-MIT Division of Health Sciences and Technology, Massachusetts Institute of Technology, Cambridge, Massachusetts, USA

[3] Mechanical Engineering Department, Massachusetts Institute of Technology, Cambridge, Massachusetts, USA

[4] Harvard Medical School, Cambridge, Massachusetts, USA

Correspondence to: hngnsong@mit.edu; hherr@media.mit.edu

Supplemental Materials

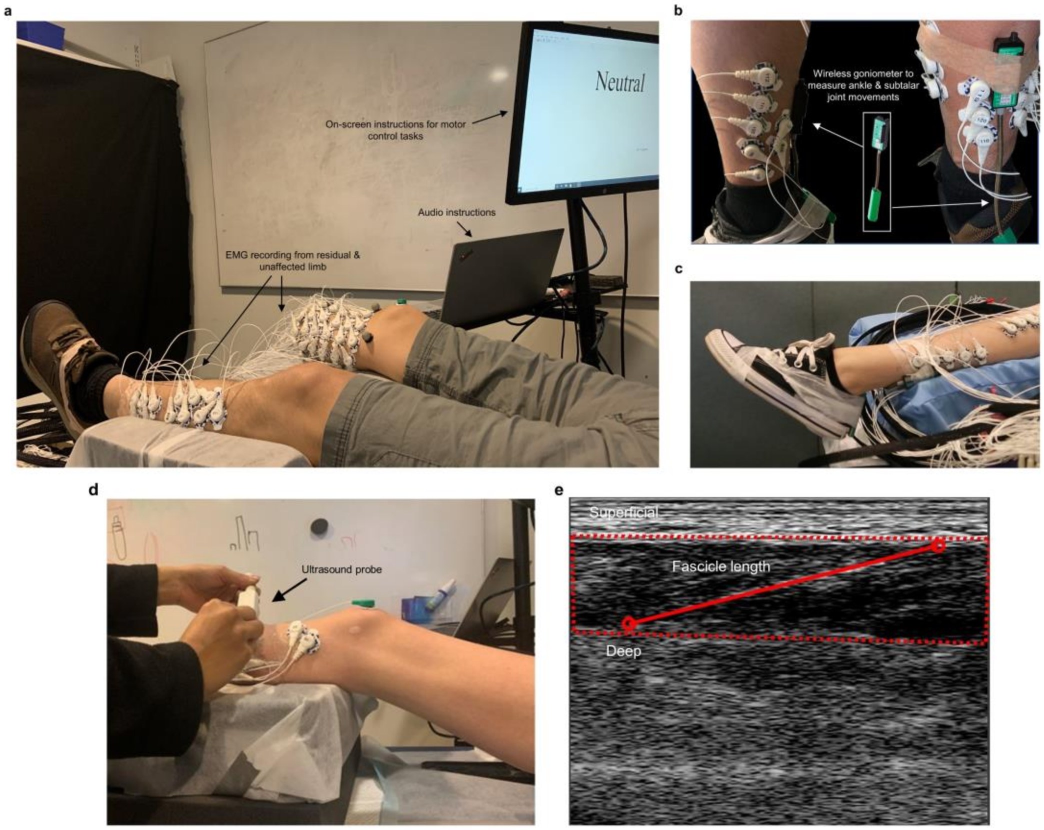

Supplementary Figure 1. **Experimental setup for clinical evaluations.** In **a**), surface electromyography (EMG) sensors and a two degrees-of-freedom goniometer were placed on the subjects' lower extremities. Each subject was then asked to repeat free-space movements of both lower extremities, with mirroring, under the guidance of both on-screen and audio instructions. Subjects were in a recumbent supine position. No visual or functional feedback of subjects' task performances was given to investigate the impact of proprioceptive sensory feedback to sensory motor responses alone. In **b**), a goniometer was placed on the posterior aspect of the intact ankle to collect mirrored phantom limb perception. In **c**), subjects' limbs were positioned to ensure comfort and the full range of ankle and subtalar movements. In **d**), an ultrasound probe was placed on the antagonist muscle to assess muscle fascicle length during cyclic agonist muscle contraction. In **e**), muscle fascicle lengths were tracked and measured using UltraTrack software (MATLAB).

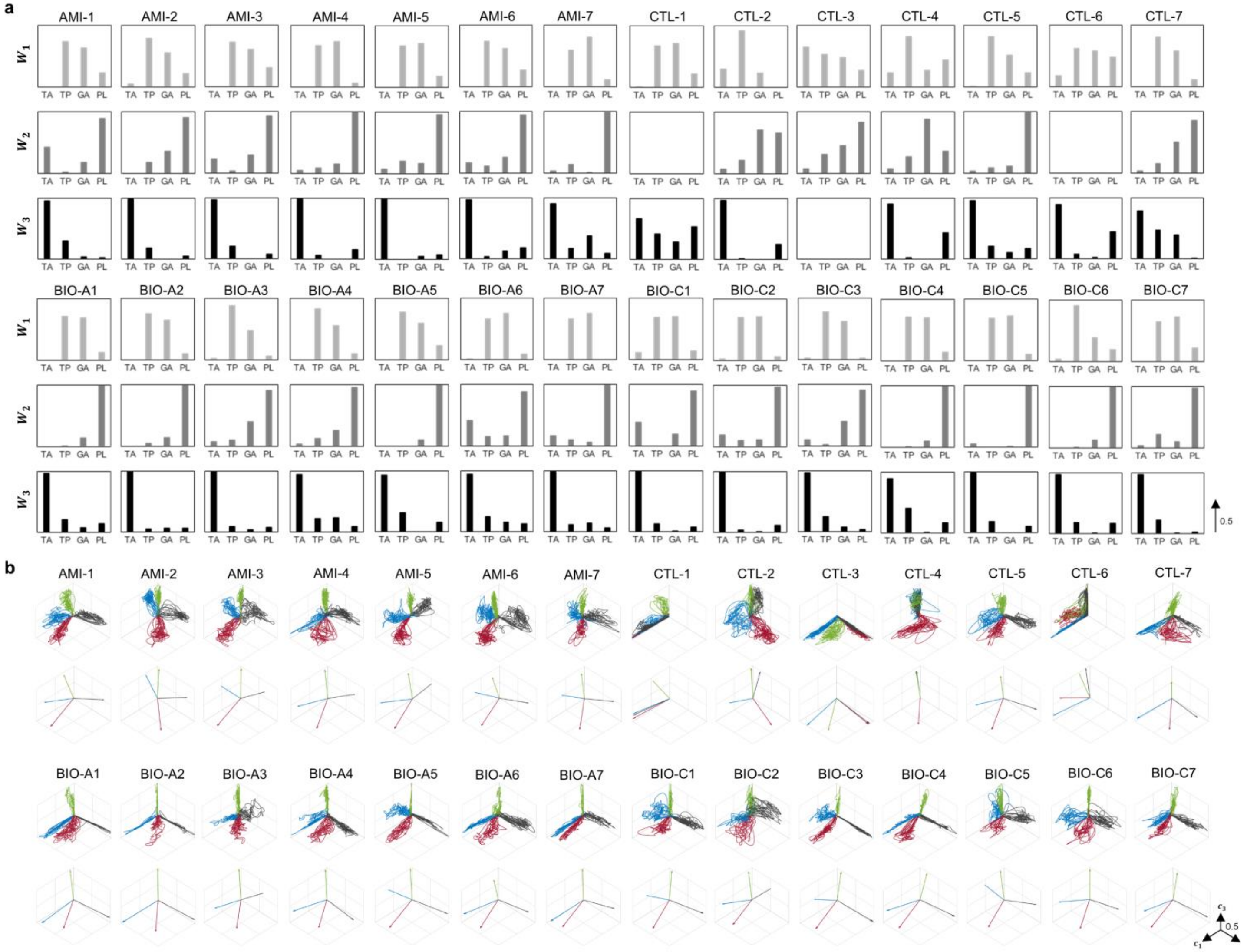

Supplementary Figure 2. Muscle synergy and synergy activation profiles are plotted including all subjects from discrete movement trials. In **a**), muscle synergies are shown, and in **b**), synergy activation vectors are plotted (AMI: n=7, CTL: n=7, BIO: n=14). Three synergy vectors were able to adequately reconstruct motor outputs of each BIO limb, all AMI subjects, and 4/7 CTL subjects (CTL-2, 4, 5, and 7). For CTL subjects 1, 3, and 6, the motor outputs were adequately reconstructed by only two synergy vectors. Common features in muscle synergy vectors of all subjects in the AMI and BIO groups were found, including strong synergy between TP and GA, and strong independent motor control of PL and TA. In the CTL group, only synergy vectors of CTL 5 shared features with those of the AMI and BIO groups. All AMI and BIO subjects demonstrated four distinguishable synergy activation vectors for the four discrete movements of ankle and subtalar joints. In the CTL group, four distinguishable synergy activation vectors were found only from CTL-2, CTL-5 and CTL-7.

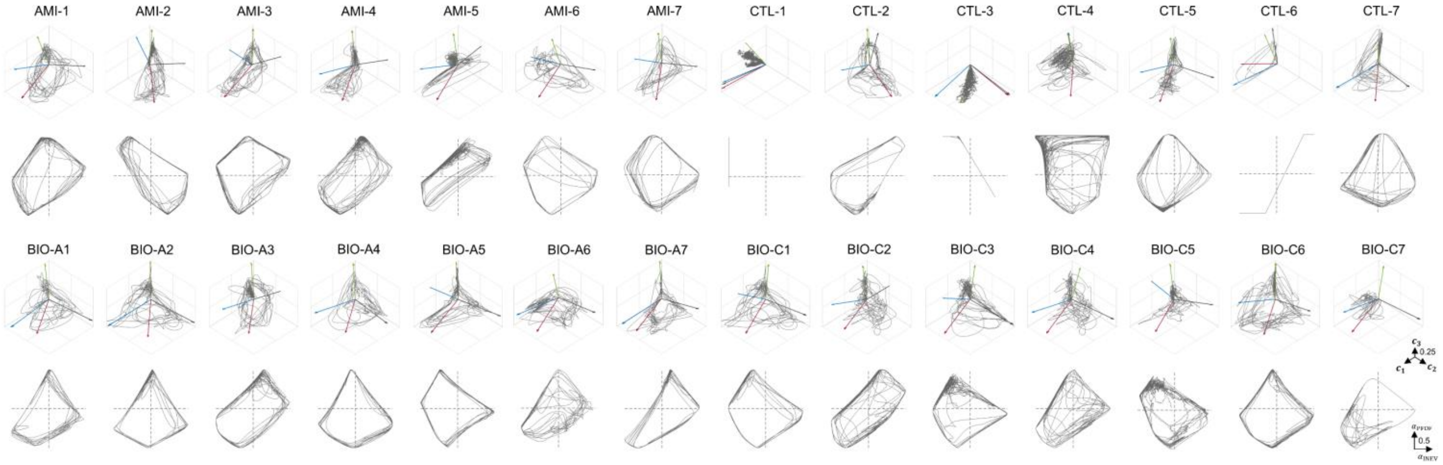

Supplementary Figure 3. Trajectories of U_S are plotted in synergy space and α-space of each subject during ankle and subtalar joint rotation trials (AMI: n=7, CTL: n=7, BIO: n=14). Three types of trajectories were found in the α-space: diamond-type, rectangle-type, and straight line. When the circle was drawn, ideally in joint space with ankle and subtalar movement transitions in concert, the directionality of motor intent in ankle and subtalar joints changed simultaneously in a coordinated manner, resulting in diamond-type trajectories consisting of diagonal directionality in α-space. However, when only a single DoF motor control was performed, the directionality of motor intent in ankle or subtalar joints changed independently, which manifested as rectangle-type trajectories, consisting of vertical and horizontal directionalities in α-space. Finally, the directionality of motor intent was shown as a straight line in α-space if motor coordination lacked sufficient DoF to produce motor intent to a second dimensionality. All subjects in the AMI and BIO groups showed diamond-type trajectories in α-space. Surprisingly, only the motor intents of CTL-2, CTL-5 and CTL-7 showed a diamond-type trajectory. The motor intent of CTL-4 was shown as rectangle-type trajectories in α-space and motor intents of CTL-1, CTL-3 and CTL-6 were expressed as straight lines.

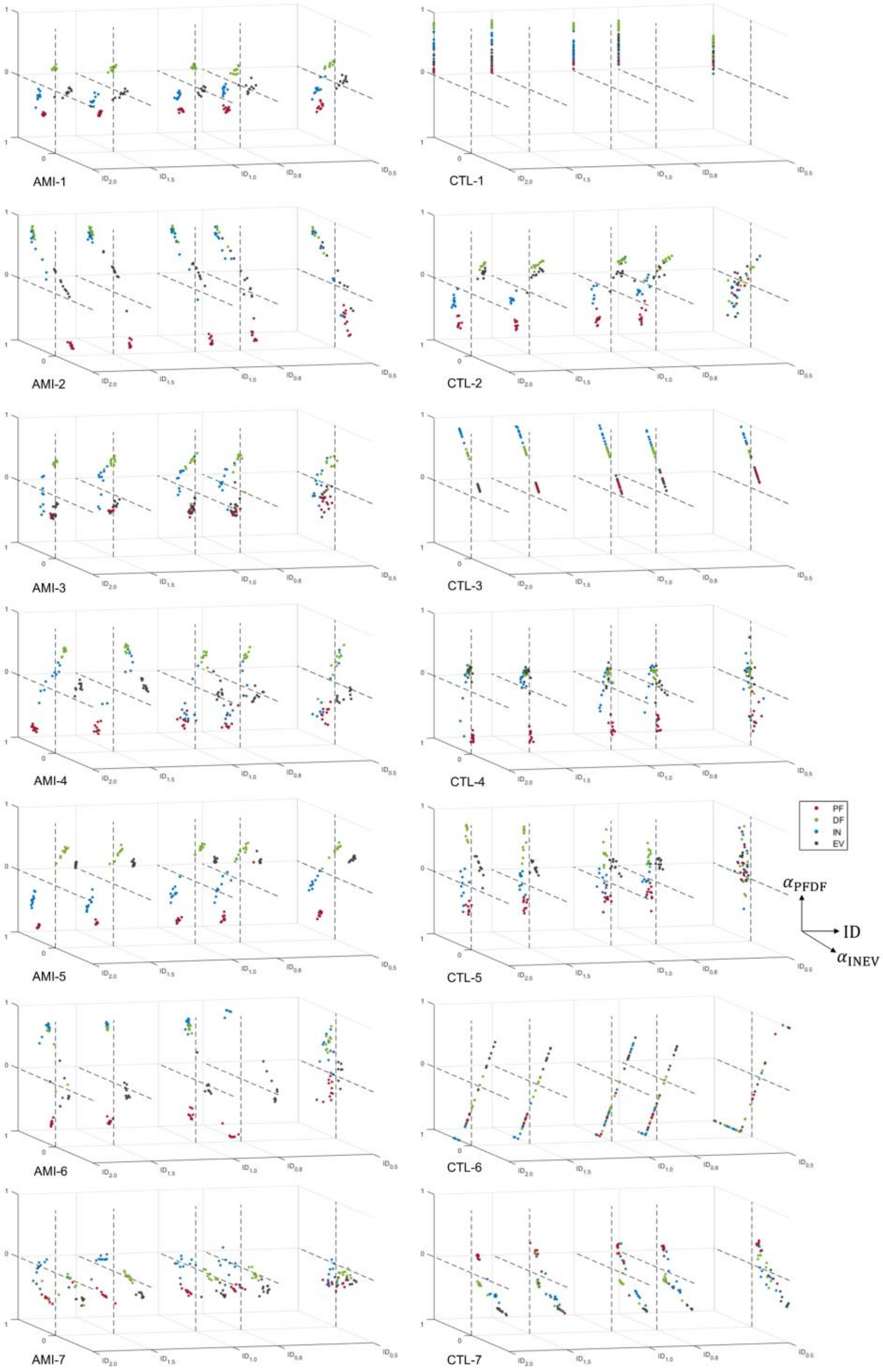

Supplementary Figure 4. Trajectories of motor intents in α-space of each subject during speed-accuracy trials (AMI: n=7, CTL: n=7). AMI-1 and 5 generated distinctive motor intents according to the given tasks for all IDs. Other AMIs were able to perform distinctive motor intents for the given task up to $\mathrm{ID}_{0.8}$ but the boundary began to decrease between motor intents at $\mathrm{ID}_{0.5}$, implying a deterioration of motor control performance. CTL-2, 4, 5, and 7 lost the boundaries of motor intent completely at $\mathrm{ID}_{0.5}$. For CTL-1, 3, and 6 motor intents were depicted as a line in each α-space, indicating the lack of DoF in motor control to perform four discrete movements.

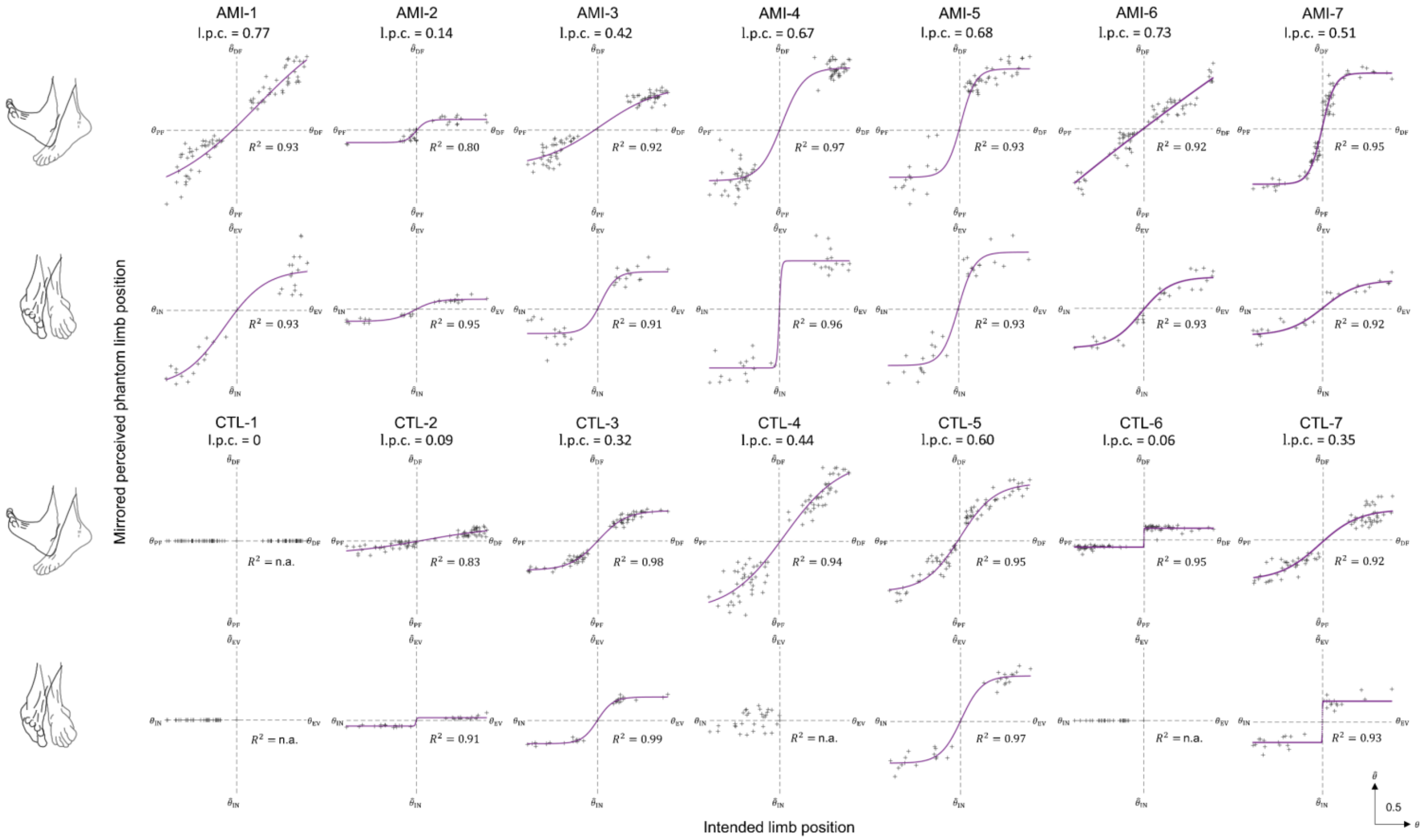

Supplementary Figure 5. Subject-specific phantom limb perception data are plotted (AMI: n=7, CTL: n=7). Limb perception capacity (l.p.c.) and R^2 of identified psychometric functions are reported. All fitted psychometric functions showed P<0.001 for the null hypothesis of the sampling mean. All AMI subjects demonstrated positional perception capacity for both phantom ankle and subtalar joint movements although AMI-2 showed limited limb perception capacity compared to the rest of the AMI subjects. CTL-2, CTL-3, CTL-5 and CTL-7 showed limb perception capacity for both ankle and subtalar joint movements. CTL-4 reported random phantom limb perception of their intended limb position for the subtalar joint movements. CTL-1 reported zero phantom limb perception. CTL-6 reported zero range of motion for the subtalar movements.

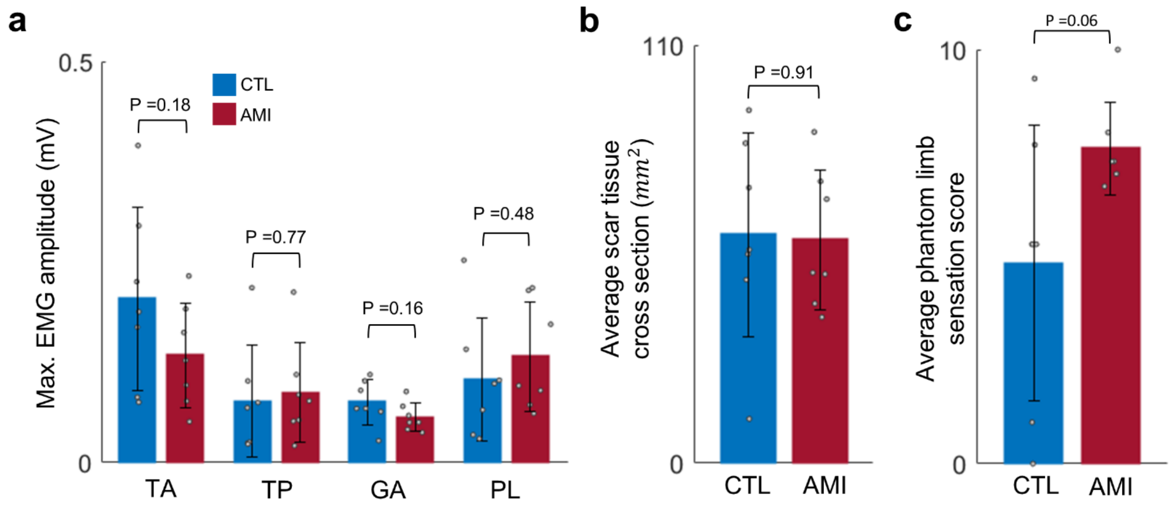

Supplementary Figure 6. Statistical analyses on clinical measures (CTL: n = 7, AMI: n = 7). In **a)**, no significant differences were found in maximum EMG amplitudes of all four muscles investigated in this study between the AMI and CTL groups. In **b)**, the average scar tissue cross sectional area within residual muscles was accessed through ultrasound imaging. No significant difference was found between the AMI and CTL groups. In **c)**, the average phantom limb score of AMI and CTL groups showed no significant difference. Unpaired two-tailed t-tests were performed for the AMI and CTL comparisons shown in subplots **a)** through **c)**. Bars and error bars represent mean and s.d., respectively.

Supplementary Table 1. Reported phantom limb sensation score and phantom limb pain. Vividness of phantom limb sensation is reported on a scale of 0 to 10, where values of 0 and 10 respectively indicate no sensation or equivalent sensations between the subject's phantom joint and their biologically intact limbs.

Subject ID	Ankle joint	Subtalar joint	Ankle/subtalar rotation	Mean score	Reported phantom limb pain
AMI-1	10	10	10	10	No serious or prohibitive pain
AMI-2	8	7	7	7.3	Tingling
AMI-3	8	8	8	8	No serious or prohibitive pain
AMI-4	8	7	7	7.3	No serious or prohibitive pain
AMI-5	7	6	7	6.7	No serious or prohibitive pain
AMI-6	9	6	6	7	No serious or prohibitive pain
AMI-7	9	5	7	7	No serious or prohibitive pain
CTL-1	0	0	0	0	Frozen phantom limb, ski boot sensation
CTL-2	9	9	10	9.3	'Firework' sensations
CTL-3	5	5	6	5.3	Pins and needle sensation, occasional itching
CTL-4	7	3	6	5.3	Numbness
CTL-5	8	7	8	7.7	No serious or prohibitive pain
CTL-6	2	0	0	1	No serious or prohibitive pain
CTL-7	6	5	5	5.3	No serious or prohibitive pain

Supplementary Table 2. Full statistics of Shapiro-Wilk, t-tests, 2x2 ANOVA, and Cohen's d.

Test	**Shapiro-Wilk test**			**Paired/unpaired t-tests**					**Effect size**
Value		W	P		s.d. or m.s.	df	t or F	P	d
**Muscle synergy similarity**	BIO-A	0.906	0.369	BIO-A:AMI	s.d. = 0.014	6	t = 0.475	0.326	1.33
	BIO-C	0.908	0.380	BIO-C:CTL	s.d. = 0.173	6	t = 3.374	0.008	
	AMI	0.900	0.333	AMI:CTL	s.d. = 0.125	12	t = 3.296	0.006	
	CTL	0.900	0.332						
				**Interactions**	m.s. = 0.083	1	F = 10.68	0.003	
**Synergy activation similarity**	BIO-A	0.952	0.750	BIO-A:AMI	s.d. = 0.021	6	t = 1.785	0.062	1.26
	BIO-C	0.857	0.141	BIO-C:CTL	s.d. = 0.139	6	t = 3.267	0.009	
	AMI	0.823	0.069	AMI:CTL	s.d. = 0.095	12	t = 2.988	0.011	
	CTL	0.936	0.604						
				**Interactions**	m.s. = 0.043	1	F = 9.22	0.006	
**2-DoF motor controllability**	BIO-A	0.897	0.311	BIO-A:AMI	s.d. = 0.034	6	t = 0.604	0.284	1.35
	BIO-C	0.957	0.794	BIO-C:CTL	s.d. = 0.256	6	t = 3.255	0.009	
	AMI	0.853	0.132	AMI:CTL	s.d. = 0.158	12	t = 3.421	0.005	
	CTL	0.940	0.639						
				**Interactions**	m.s. = 0.165	1	F = 12.22	0.002	
