# Supplementary material for: Agonist-antagonist muscle strain in the residual limb preserves motor control and perception after amputation
Source: Commun Med (Lond). 2022 Aug 5;2:97. doi: 10.1038/s43856-022-00162-z (PMC9356003; doi:10.1038/s43856-022-00162-z)
Supplement: Supplementary file 7 — Reporting Summary [file 43856_2022_162_MOESM7_ESM.pdf]

nature portfolio

Corresponding author(s): Hyungeun Song, Hugh Herr

Last updated by author(s): Jun 29, 2022

Reporting Summary

Nature Portfolio wishes to improve the reproducibility of the work that we publish. This form provides structure for consistency and transparency in reporting. For further information on Nature Portfolio policies, see our Editorial Policies and the Editorial Policy Checklist.

Statistics

For all statistical analyses, confirm that the following items are present in the figure legend, table legend, main text, or Methods section.

n/a	Confirmed	
☐	☒	The exact sample size (n) for each experimental group/condition, given as a discrete number and unit of measurement
☐	☒	A statement on whether measurements were taken from distinct samples or whether the same sample was measured repeatedly
☐	☒	The statistical test(s) used AND whether they are one- or two-sided<br>*Only common tests should be described solely by name; describe more complex techniques in the Methods section.*
☐	☒	A description of all covariates tested
☐	☒	A description of any assumptions or corrections, such as tests of normality and adjustment for multiple comparisons
☐	☒	A full description of the statistical parameters including central tendency (e.g. means) or other basic estimates (e.g. regression coefficient) AND variation (e.g. standard deviation) or associated estimates of uncertainty (e.g. confidence intervals)
☐	☒	For null hypothesis testing, the test statistic (e.g. F, t, r) with confidence intervals, effect sizes, degrees of freedom and P value noted<br>*Give P values as exact values whenever suitable.*
☒	☐	For Bayesian analysis, information on the choice of priors and Markov chain Monte Carlo settings
☒	☐	For hierarchical and complex designs, identification of the appropriate level for tests and full reporting of outcomes
☐	☒	Estimates of effect sizes (e.g. Cohen's d, Pearson's r), indicating how they were calculated

Our web collection on statistics for biologists contains articles on many of the points above.

Software and code

Policy information about availability of computer code

Data collection	EMG data were collected for 12 of the 14 subjects (6 AMI, 6 CTL) using a TMSi Refa 136 Physiological Signal Amplifier (Twente Medical Systems International, Netherlands). EMG data were collected for the other 2 subjects (1 AMI, 1 CTL) using a Wireless Delsys Trigno (Delsys, Natick, MA USA). The mirrored phantom limb sensations data were collected using a Wireless Twin-axis Goniometer (Biometrics ltd, UK). Fascicle strain was recorded using a high-definition real-time ultrasound scanner (LS128, Telemed, Lithuania).
Data analysis	All data analyses were performed using custom codes, written on MATLAB 2020b (Mathworks, USA). The code related to this study is available at https://doi.org/10.5281/zenodo.6534101. The fascicle strain was estimated from ultrasound video recordings using the Ultra Track V2 MATLAB package.

For manuscripts utilizing custom algorithms or software that are central to the research but not yet described in published literature, software must be made available to editors and reviewers. We strongly encourage code deposition in a community repository (e.g. GitHub). See the Nature Portfolio guidelines for submitting code & software for further information.

Data

Policy information about availability of data

All manuscripts must include a data availability statement. This statement should provide the following information, where applicable:

- Accession codes, unique identifiers, or web links for publicly available datasets
- A description of any restrictions on data availability
- For clinical datasets or third party data, please ensure that the statement adheres to our policy

All data associated with this study are found in the main text and the Supplementary Materials. Source data underlying the main figures in the manuscript are

available as Supplementary Data 1-3 and Supplementary Figure 5. Individual participant data are not available due to limitations imposed by the informed consent form signed by the researcher team and study participants in advance of data collection.

Field-specific reporting

Please select the one below that is the best fit for your research. If you are not sure, read the appropriate sections before making your selection.

☒ Life sciences ☐ Behavioural & social sciences ☐ Ecological, evolutionary & environmental sciences

For a reference copy of the document with all sections, see nature.com/documents/nr-reporting-summary-flat.pdf

Life sciences study design

All studies must disclose on these points even when the disclosure is negative.

Sample size	No statistical methods were used to pre-determine sample size. The effect sizes of Cohen's d values from main results varied between 1.26-to-1.35.
Data exclusions	No data were excluded from the analyses
Replication	All motor control and proprioceptive perception experiments successfully showed consistent results in fourteen independent amputees.
Randomization	-During the identification procedures of motor coordination, discrete ankle and subtalar joint movements were not randomized to ensure amputee's optimal motor control performance.<br>-The rotations of ankle and subtalar joints were not applicable for the randomization.<br>-The assessments of spatiotemporal motor control and phantom limb perception were performed by randomizing the sequence of the motor control tasks.
Blinding	The investigators were not blinded to tested conditions. However, the analyses were conducted in both group comparison and amputee-specific features.

Reporting for specific materials, systems and methods

We require information from authors about some types of materials, experimental systems and methods used in many studies. Here, indicate whether each material, system or method listed is relevant to your study. If you are not sure if a list item applies to your research, read the appropriate section before selecting a response.

Materials & experimental systems

n/a	Involved in the study	
☒	☐	Antibodies
☒	☐	Eukaryotic cell lines
☒	☐	Palaeontology and archaeology
☒	☐	Animals and other organisms
☐	☒	Human research participants
☐	☒	Clinical data
☒	☐	Dual use research of concern

Methods

n/a	Involved in the study	
☒	☐	ChIP-seq
☒	☐	Flow cytometry
☒	☐	MRI-based neuroimaging

Human research participants

Policy information about studies involving human research participants

Population characteristics	Fourteen individuals with unilateral transtibial acute amputations participated in the study. Seven of these subjects had undergone an Agonist-antagonist Myoneural Interface (AMI) amputation (AMI group) and seven subjects had undergone non-AMI amputation (CTL group). All subjects were tested within 6 months to 8.7 years of their amputation surgery. Subject age ranged from 25 to 62 years old at the time they were tested, with average and s.d. of 45 ± 14 years. Study populations for the AMI group and the CTL group were matched to the degree possible for age, time since amputation, and body habitus, although there were no formal exclusion criteria in this regard. The ratio of female to male subjects was 5:2.
Recruitment	AMI group subjects were drawn from the pool of patients who had undergone an AMI amputation procedure at Brigham and Women's Hospital (BWH) in Boston MA under Partners Healthcare IRB protocol P2014001379. CTL group subjects were recruited by word of mouth or external inquiries. Potential subjects spoke to a team member who described the study, answered questions, and provided links to the Clinicaltrials.gov website (NCT 03913273). Individuals who decided to participate signed Informed Consent Forms at Massachusetts Institute of Technology (MIT) in Cambridge MA. Study data were collected under approval of MIT's Committee on the Use of Humans as Experimental Subjects (protocol 1812634918). All subjects met the following inclusion criteria: Age within the range of 18 years to 65 years; AMI transtibial amputation (AMI

	group) or Non-AMI transtibial amputation (CTL group); Fully healed amputation site, Proficiency in using a standard lower extremity prosthesis, and Activity or K-Level of at least K3 to K4 (capability to ambulate with variable cadence).
Ethics oversight	The study protocol was approved by the MIT Committee on the Use of Humans as Experimental Subjects, which is MIT's IRB.

Note that full information on the approval of the study protocol must also be provided in the manuscript.

Clinical data

Policy information about clinical studies

All manuscripts should comply with the ICMJE guidelines for publication of clinical research and a completed CONSORT checklist must be included with all submissions.

Clinical trial registration	The ClinicalTrials.gov Registration for this work is NCT03913273.
Study protocol	Information about the clinical trial study protocol NCT03913273, which has as a secondary ID R01HD097135, is publicly accessible at https://clinicaltrials.gov/ct2/show/NCT03913273. The study record was first posted in April 2019 and is regularly updated, most recently December 2021.
Data collection	All data were collected at the Massachusetts Institute of Technology (MIT). The time period of recruitment and data collection were from June 12, 2019 through September 19, 2021.
Outcomes	The present work begins to investigate one outcome measure – economy of motion – of our ongoing clinical trial, NCT03913273, although we do not report any pre-specified endpoints of that trial in the present work. The relationship of the present work to that trial is to obtain preliminary data and an algorithmic framework – a muscle synergy model – and thus inform our assessment of our pre-specified outcome measures for NCT03913273.

March 2021
